# Lachesis to the Rescue

**Published:** 1896-01

**Authors:** Jos. Fitz-Mathew

**Affiliations:** Victoria, Penna.


### LACHESIS TO THE RESCUE.

Jos. Fitz-Mathew, M. D., Victoria, Penna.

Mrs P., æt. thirty-six years; bilious temperament; inclined to be hypochondriacal; had grippe during the winter of 1894. (Allopathic treatment.) As a sequel, she was attacked by violent neuralgic pains in left shoulder and arm, which resisted all treatment up to the month of May, 1895, when I was requested to prescribe, and, in a hasty interview upon the eve of a journey, obtained the following history and symptoms: Mrs. P. was a school-teacher, and was suffering from "too much mother-in-law;" a captious person, holding the family purse-strings; an expert in the art of nagging. Sharp, darting pains, starting from the cervical region, extending through the left shoulder down the left arm and into the fingers at times. Pain worse in upper arm, with a sensation as if it would burst. Intermissions at night, during which patient slept well. I assumed this to be a case of cervico-brachial neuralgia, without any examination. Considering the mental conditions and the pains running down to the fingers, I prescribed—with little confidence, however—Sepia$^{2c}$ (D.), which produced no appreciable effect. Subsequently several other remedies were tried with like result, and I felt much mortified at being unable to ellicit from the patient any guiding key-note symptom for the selection of the proper remedy. Having been recommended to this patient by a friend, who had expressed much confidence in the result of homœopathic treatment in my hands, I was especially disgusted with my failure, and the probable relapse of this patient to allopathic treatment. In response to my earnest solicitation for more symptoms, I received the following: "Suffered severe pain all night, and this morning it is no better. I am sure it will be serious if it continues much longer. My left arm is wasting, getting smaller. It seems to me that my patience and endurance are exhausted. I am so discouraged and disheartened. Is it alarming? I feel well otherwise, but with this constant nagging pain in elbow, shoulder, chest, etc., I feel quite worried. It hurts both front and back to cough. I have a great deal of palpitation and pain in the left side. Do you think my heart is affected? If this pain lasts much longer, I don't care to live. Will my arm ever be restored to its natural size? * * * Pain still very severe, and on rising I have a feeling in the middle of the stomach *as if something was rolling and bounding about.*"

(Patients usually refer to the abdomen as the stomach.) The patient was now given one dose of Lachesis$^{cm}$ (Fincke), with Sac-lac. ad lib., and assured that the left arm was not wasting away, and that it should be measured to convince her of the fact. The next letter says: "I am feeling much better, having had very little pain, but there is a severe twitching of the muscles where the pain was." Sent Sac-lac. Subsequently a slight return of the pain in the arm induced me to send another dose of Lachesis$^{cm}$. After this I received the following: "I have had no sign of the old trouble for some time, and feel much better; but I have such a sore pain at the very lowest extremity of the spine (coccyx). It is sore to touch; it hurts me to sit, and sometimes to walk. I am afraid some growth may be forming there." Sent Sac-lac. and assured the patient *this* pain would subside. Measurement of the left arm showed no appreciable diminution. This patient received—as shown—two doses of Lachesis$^{cm}$ (Fincke), and is quite free from her troubles in respect to the pains.

